# Ambient Air Quality Standards and Policies in Eastern Mediterranean Countries: A Review

**DOI:** 10.3389/ijph.2023.1605352

**Published:** 2023-02-20

**Authors:** Sasan Faridi, Michal Krzyzanowski, Aaron J. Cohen, Mazen Malkawi, Heba Adel Moh’d Safi, Fatemeh Yousefian, Faramarz Azimi, Kazem Naddafi, Fatemeh Momeniha, Sadegh Niazi, Heresh Amini, Nino Künzli, Mansour Shamsipour, Adel Mokammel, Vahid Roostaei, Mohammad Sadegh Hassanvand

**Affiliations:** ^1^ Center for Air Pollution Research (CAPR), Institute for Environmental Research (IER), Tehran University of Medical Sciences, Tehran, Iran; ^2^ Department of Environmental Health Engineering, School of Public Health, Tehran University of Medical Sciences, Tehran, Iran; ^3^ Environmental Research Group, School of Public Health, Imperial College London, London, United Kingdom; ^4^ Institute for Health Metrics and Evaluation, University of Washington, Seattle, WA, United States; ^5^ Boston University School of Public Health, Boston, MA, United States; ^6^ Health Effects Institute, Boston, MA, United States; ^7^ World Health Organization/Regional Office of the Eastern Mediterranean/Climate Change, Health and Environment Unit (WHO/EMR/CHE), Amman, Jordan; ^8^ Department of Environmental Health Engineering, Faculty of Health, Kashan University of Medical Sciences, Kashan, Iran; ^9^ Environmental Health Research Center, School of Health and Nutrition, Lorestan University of Medical Sciences, Khorramabad, Iran; ^10^ Center for Solid Waste Research, Institute for Environmental Research (IER), Tehran University of Medical Sciences, Tehran, Iran; ^11^ International Laboratory for Air Quality and Health, Faculty of Science, School of Earth and Atmospheric Sciences, Queensland University of Technology (QUT), Brisbane, QLD, Australia; ^12^ Department of Public Health, University of Copenhagen, Copenhagen, Denmark; ^13^ Swiss Tropical and Public Health Institute, Allschwil, Switzerland; ^14^ University of Basel, Basel, Switzerland; ^15^ Department of Research Methodology and Data Analysis, Institute for Environmental Research (IER), Tehran University of Medical Sciences, Tehran, Iran

**Keywords:** air pollution, Eastern Mediterranean Region, air quality standards, air quality guidelines, NAAQS

## Abstract

**Objectives:** National ambient air quality standards (NAAQS) are critical tools for controlling air pollution and protecting public health. We designed this study to 1) gather the NAAQS for six classical air pollutants: PM_2.5_, PM_10_, O_3_, NO_2_, SO_2_, and CO in the Eastern Mediterranean Region (EMR) countries, 2) compare those with the updated World Health Organizations Air Quality Guidelines (WHO AQGs 2021), 3) estimate the potential health benefits of achieving annual PM_2.5_ NAAQS and WHO AQGs per country, and 4) gather the information on air quality policies and action plans in the EMR countries.

**Methods:** To gather information on the NAAQS, we searched several bibliographic databases, hand-searched the relevant papers and reports, and analysed unpublished data on NAAQS in the EMR countries reported from these countries to the WHO/Regional office of the Eastern Mediterranean/Climate Change, Health and Environment Unit (WHO/EMR/CHE). To estimate the potential health benefits of reaching the NAAQS and AQG levels for PM_2.5_, we used the average of ambient PM_2.5_ exposures in the 22 EMR countries in 2019 from the Global Burden of Disease (GBD) dataset and AirQ+ software.

**Results:** Almost all of the EMR countries have national ambient air quality standards for the critical air pollutants except Djibouti, Somalia, and Yemen. However, the current standards for PM_2.5_ are up to 10 times higher than the current health-based WHO AQGs. The standards for other considered pollutants exceed AQGs as well. We estimated that the reduction of annual mean PM_2.5_ exposure level to the AQG level (5 μg m^−3^) would be associated with a decrease of all natural-cause mortality in adults (age 30+) by 16.9%–42.1% in various EMR countries. All countries would even benefit from the achievement of the Interim Target-2 (25 μg m^−3^) for annual mean PM_2.5_: it would reduce all-cause mortality by 3%–37.5%. Less than half of the countries in the Region reported having policies relevant to air quality management, in particular addressing pollution related to sand and desert storms (SDS) such as enhancing the implementation of sustainable land management practices, taking measures to prevent and control the main factors of SDS, and developing early warning systems as tools to combat SDS. Few countries conduct studies on the health effects of air pollution or on a contribution of SDS to pollution levels. Information from air quality monitoring is available for 13 out of the 22 EMR countries.

**Conclusion:** Improvement of air quality management, including international collaboration and prioritization of SDS, supported by an update (or establishment) of NAAQSs and enhanced air quality monitoring are essential elements for reduction of air pollution and its health effects in the EMR.

## Introduction

Outdoor (ambient) air pollution is a major environmental health risk factor affecting people all over the world (1–4). The World Health Organization (WHO) reported that 99% of the world population in 2019 was living in places where the updated WHO Air Quality Guidelines (AQG) level for annual average fine particular matter (PM_2.5_) concentration (5 μg m^−3^) was not met (5). It has also been reported that nearly 95% of the world’s population in 2016 lived in areas with ambient PM_2.5_ concentrations exceeding the 2005 WHO AQG level (10 μg m^−3^), particularly in the EMR countries (1, 6–8). The underlying reasons of high annual ambient PM_2.5_ concentrations across the East-Mediterranean Region (EMR) countries are associated with unsustainable development, continuing urbanization and industrialization, increasing emissions from mobile sources, as well as sand and dust storm (SDS) events. Air pollution abatement policies, existing in some countries of the region, are not efficient enough to cope with the pollution (9–12). Ambient PM_2.5_ air pollution was the 6^th^ leading mortality risk factor in the region in 2019, contributing to 389 (uncertainty interval 320-465) thousand deaths in EMR countries. This represents a considerable increase since 1990 when ambient PM_2.5_ was the 11th leading mortality risk factor, accounting for 159 (117-215) thousand deaths (13). By reducing ambient air pollution levels, particularly PM_2.5_, to the updated WHO AQGs level, nations in the EMR with a population of nearly 680 million people could reduce the burden of disease attributed to air pollution by 81% (5).

**FIGURE 1:**
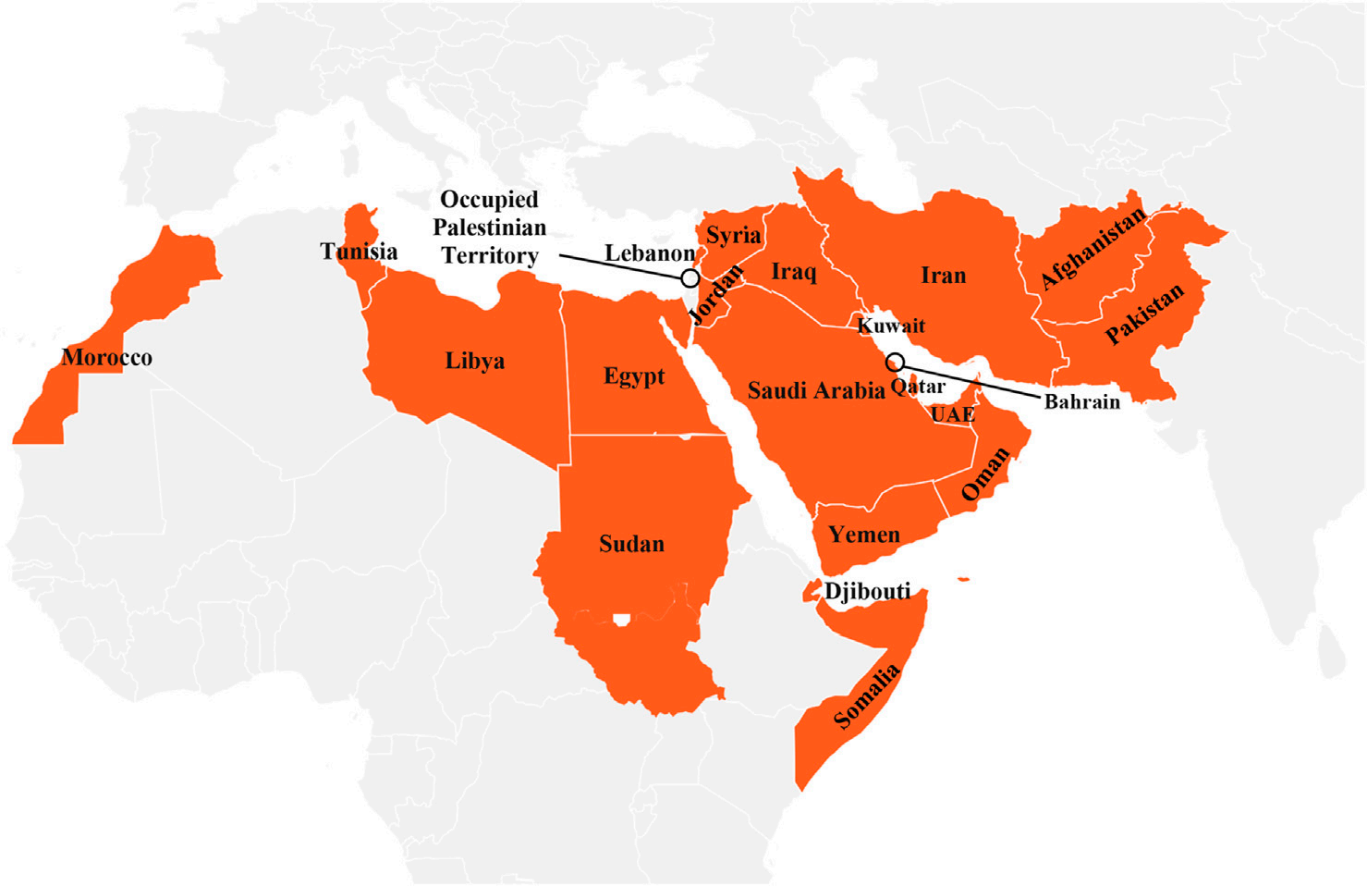
Spatial distribution of 22 Eastern Mediterranean Region countries (Eastern Mediterranean Region. 2022).

The aim of this study is to assess the EMR countries (Figure 1) legal and organizational capacities for air pollution abatement. In particular, we aimed to 1) gather the information on NAAQS in the EMR countries, 2) compare those standard levels with the updated WHO AQGs and interim targets (ITs), 3) estimate potential health benefits of achieving annual PM_2.5_ national ambient air quality standards (NAAQS) and WHO AQGs per country, and 4) gather the information on air quality policies and action plans in the EMR countries, in particular those related to SDS as one of the most important sources affecting ambient air quality of the EMR countries (14). Evaluation of this information allows us to formulate conclusions on the steps necessary for air quality improvement and reduction of the burden of air pollution on population health in the region.

## Methods

To gather the information on published and unpublished NAAQS in the EMR countries, several approaches were used as follows: first, we conducted a systematic search of the articles according to the Preferred Reporting and Items for Systematic Review and Meta-Analysis (PRISMA) criteria (15, 16), as shown in the Supplementary Figure S1. The search was performed on 25 January 2022. To access the relevant studies, we queried three English language databases, including Scopus, PubMed, and Web of Science Core Collection (WOS) using the following search keywords: “national ambient air quality standards” and “ambient air quality standards.” Second, the Google Scholar database for papers and reports published in English from the database inception until 25 January 2022, was searched using the search terms “ambient air quality standards” and “WHO air quality guidelines.” Third, to increase the sensitivity and gather more relevant records, additional documents were identified from hand-searching in the relevant papers and reports identified through Google Scholar database. For the relevant studies, we considered the papers which, according to the title, specifically focused on air quality standards or guidelines. Fourth, we received the reports of conference of Gulf Cooperation Council (GCC) countries (10–11 January 2022) from the WHO/EMRO/CHE. Fifth, we designed and used a questionnaire: “A questionnaire to collect health, population, and air quality monitoring and management data in the Eastern Mediterranean Region” to collect the detailed information regarding the ambient air quality management strategy/framework/plan of action (AQAP) in the EMR countries (Supplementary Questionnaire S1). The questionnaire included sections on NAAQS, ambient air quality monitoring network, unpublished source apportionment/emission inventory studies, availability and accessibility of health-based data, information release and public participation, and human resources and institutional capabilities relevant for actions on ambient air pollution. It should be highlighted that only information concerning AQAP and NAAQS has been reported in this article. This questionnaire was distributed in the period from September 2021 to April 2022 by the WHO/EMRO/CHE colleagues to the national air quality and health experts of the EMR countries through WHO Country Offices. We have also reviewed the WHO Air Quality Database (version from March 2022, https://www.who.int/data/gho/data/themes/air-pollution/who-air-quality-database) to assess the availability and accessibility of data on PM_2.5_, PM_10_, and NO_2_ concentrations from air quality monitoring in the EMR cities in 2015–2020.

To estimate the potential health benefits of reducing PM_2.5_ exposure to achieve annual PM_2.5_ NAAQS, WHO AQG level or its Interim Targets (ITs) in each country (Table 1), we applied WHO AirQ+ (v.2.1) software (https://www.who.int/europe/tools-and-toolkits/airq---software-tool-for-health-risk-assessment-of-air-pollution). We calculated population attributable fraction (PAF) using the population-weighted mean exposure to PM_2.5_ in 2019 in each country from the GBD project (17) as well as the log-linear concentration-response function with relative risk for all natural-cause mortality in adults of 1.08 (95% C.I. 1.06–1.09) per 10 μg m^−3^ from the meta-analysis of Chen J. and Hoek. G, 2020(18). PAF is the proportion of current adult mortality attributable to exposure exceeding a certain, lower than currently observed, level (here: NAAQS, Its, or AQG level). We have not calculated the number of deaths or mortality attributed to the excess exposure since relevant age-specific mortality and population data from each of the countries were not available to us.

**TABLE 1:** Potential health benefits of achieving annual fine particulate matter (PM_2.5_) National ambient air quality standards, World Health Organization Air Quality Guidelines, and Interim Targets per country based on the population attributable fraction (Eastern Mediterranean Region. 2022).

Country	Average annual (2019) population-weighted PM_2.5_ (µg m^−3^)^a^	Percent of all natural cause mortality (PAF in %)^b^ attributed to pollution exceeding						PAF (NAAQS)/PAF (AQG) (%)
		NAAQS	IT-1 (35 μg m^−3^)	IT-2 (25 μg m^−3^)	IT-3 (15 μg m^−3^)	IT-4 (10 μg m^−3^)	WHO AQG (5 μg m^−3^)	
Afghanistan	52.4	12.5 (9.6–13.9)	12.5 (9.6–13.9)	19 (14.8–21)	25 (19.6–27.6)	27.8 (21.9–30.6)	30.6 (24.1–33.5)	41
Bahrain	59.2	23.1 (18.1–25.5)	17 (13.1–18.8)	23.1 (18.1–25.5)	28.8 (22.7–31.7)	31.5 (24.9–34.6)	34.1 (27.1–37.1)	68
Djibouti	43.2	—	6.1 (4.7–6.8)	13.1 (10.1–14.5)	19.5 (15.1–21.6)	22.6 (17.6–24.9)	25.5 (19.9–28.1)	—
Egypt	67.9	12.9 (9.9–14.3)	22.4 (17.4–24.7)	28.1 (22.1–30.9)	33.4 (26.5–36.3)	35.9 (28.6–39.3)	38.4 (30.7–41.8)	34
Iran	38	18.1 (14.1–20.1)	2.3 (1.7–2.6)	9.5 (7.3–10.6)	16.2 (12.5–17.9)	19.4 (15.1–21.4)	22.4 (17.5–24.8)	81
Iraq	48.5	25.6 (20.1–28.2)	9.9 (7.6–10.9)	16.5 (12.8–18.3)	22.7 (17.7–25.1)	25.6 (20.1–28.2)	28.5 (22.4–31.3)	90
Jordan	30.6	11.3 (8.7–12.6)	—	4.2 (3.2–4.7)	11.3 (8.7–12.6)	14.7 (11.3–16.3)	17.9 (13.8–19.8)	63
Kuwait	61	—	18.1 (14.1–20)	24.2 (19–26.7)	29.8 (23.5–32.7)	32.5 (25.7–35.6)	35 (27.8–38.3)	—
Lebanon	29	—	—	3 (2.3–3.4)	10.2 (7.8–11.4)	13.6 (10.5–15.1)	16.9 (13–18.7)	—
Libya	38.6	—	2.7 (2.1–3.1)	9.9 (7.6–11.1)	16.6 (12.8–18.4)	19.8 (15.4–21.8)	22.8 (17.8–25.1)	—
Morocco	35.1	—	—	7.5 (5.7–8.3)	14.3 (11.1–15.9)	17.6 (13.6–19.5)	20.7 (16.1–22.8)	—
Oman	44.6	—	7.1 (5.4–7.9)	14 (10.8–15.5)	20.4 (15.8–22.5)	23.4 (18.3–25.8)	26.3 (20.6–28.9)	—
Pakistan	62.6	30.7 (24.2–33.6)	19.1 (14.9–21.2)	25.1 (19.7–27.7)	30.7 (24.2–33.7)	33.3 (26.4–36.5)	35.8 (28.5–39.1)	86
Palestine	31.3	—	—	4.7 (3.6–5.3)	11.8 (9.1–13.1)	15.1 (11.7–16.7)	18.3 (14.2–20.3)	—
Qatar	76	—	27.1 (21.3–29.8)	32.5 (25.7–35.6)	37.5 (29.9–40.8)	39.8 (31.9–43.4)	42.1 (33.9–45.8)	—
Saudi Arabia	61.5	30.1 (23.7–33)	18.5 (14.3–20.4)	25.5 (19.2–27)	30.1 (23.7–33)	32.7 (25.9–35.9)	35.3 (28.1–38.6)	85
Somalia	30.4	—	—	4.1 (3.1–4.6)	11.2 (8.6–12.4)	14.5 (11.2–16.1)	17.8 (13.8–19.7)	—
Sudan	54.7	29.1 (22.9–31.9)	14.1 (10.8–15.6)	20.4 (15.9–22.6)	26.3 (20.7–28.9)	29.1 (22.9–32)	31.8 (25.1–34.8)	92
Syria	31	—	—	4.5 (3.4–5)	11.6 (8.9–12.9)	14.9 (11.5–16.6)	18.1 (14.1–20.1)	—
Tunisia	30.4	—	—	4.1 (3.1–4.6)	11.2 (8.6–12.4)	14.5 (11.2–16.1)	17.8 (13.8–19.7)	—
UAE^c^	43.7	—	6.5 (4.9–7.2)	13.4 (10.3–14.9)	19.8 (15.4–21.9)	22.9 (17.8–25.2)	25.8 (20.2–28.4)	—
Yemen	44.5	12.5 (9.6–13.9)	7 (5.4–7.8)	13.9 (10.7–15.5)	20.3 (15.8–22.5)	23.3 (18.2–25.7)	26.2 (20.6–28.8)	—

^a^ Estimated by GBD project (https://www.stateofglobalair.org/data/#/air/plot).

^b^ In brackets: uncertainty of PAF associated with 95% C.I. of RR.

^c^ United Arab Emirates (UAE).

## Results and Discussion

### Findings on NAAQS

#### Search Results and Description of Included Papers and Reports

**FIGURE 2:**
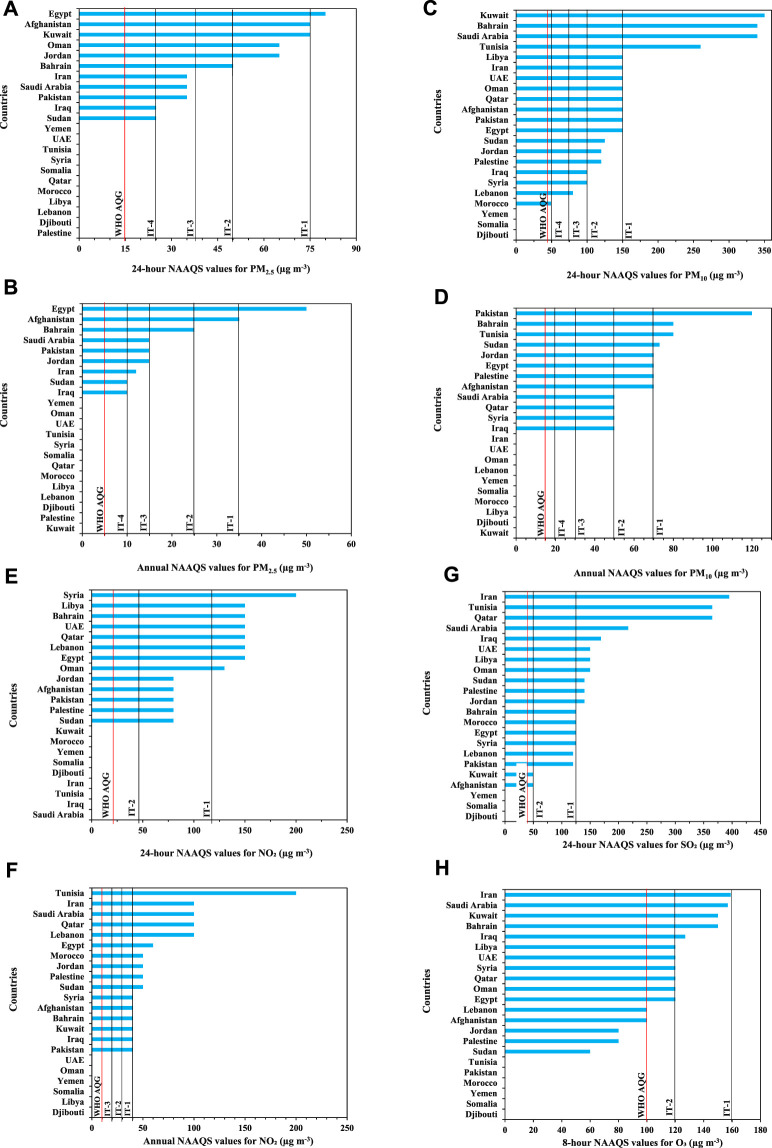
National ambient air quality standards **(A–H)** for criteria air pollutants in the Eastern Mediterranean Region countries compared to the World Health Organization Air Quality Guidelines and Interim Targets (25) (Eastern Mediterranean Region. 2022).

Out of the 1083 records identified by searching in Scopus, PubMed, WOS and Google Scholar database, only five fulfilled the search criteria and were selected for further evaluation as shown in Supplementary Figure S1. Two papers have been published on the NAAQS at global scale, while three others have been quoted in the WHO AQGs publication (19–21). Of those papers that were global in scale, we used one (21) as it had reported the standard levels for each classical air pollutant. According to this paper, 11 countries (out of 22, including the Occupied Palestine Territory as a country, http://www.emro.who.int/countries.html) in the EMR have set a standard for at least one ambient air pollutant and averaging time, one had no standards and no relevant information was available for 9 countries. Additional documents used to identify NAAQS in the EMR countries were found in the UNEP (2021) publication (22), resulting from the UNEP project conducted in 2015 (Supplementary Table S2). Questionnaires from the WHO/CHE survey provided additional information. In the result of this search, we have identified NAAQS in 19 out of 22 EMR countries (Figure 2; Supplementary Table S3). We found no information on NAAQS for Djibouti, Somalia, and Yemen. NAAQS levels vary substantially (up to a factor of 2–3) between the countries and not in all countries standards for all pollutants or averaging times are set, especially for PM_2.5_. The allowed frequency (number of days per year) of the 24-hr standard levels for PM_2.5_ or PM_10_ exceedance varies as well (Supplementary Table S3): for Afghanistan, Iran, Jordan, Kuwait, Pakistan and Saudi Arabia, the standard levels for both PM_2.5_ and PM_10_ can be exceeded 18, 4, 3, 3, 7 and 12 days/year, respectively; for Palestine and Sudan, the 24-hr standard levels for PM_10_ can be exceeded 3 days/year and 3 days/month, respectively.

#### NAAQS in the EMR Countries Compared to the Updated WHO AQGs

Figures 2A–H presents the comparison of the NAAQS for the classical ambient air pollutants in the EMR countries with the updated WHO AQGs and ITs. All NAAQS values in the EMR countries were significantly higher than those for the updated WHO AQGs and one or two of ITs. In the 11 countries which have set a 24-hour standard for PM_2.5_, their values were approximately 2–5 times higher than the WHO AQG level and for 9 of the 11 countries were equal to the IT-4 (Figure 2A). For annual PM_2.5_ mean (Figure 2B), the NAAQS was available in 9 countries and its value was 2–10 times higher than the WHO AQG level. Information on the NAAQS and their relationship to the WHO recommendations for other classical ambient air pollutants is presented in Figures 2C–H.

#### Potential Health Benefits of Achieving Annual PM_2.5_ NAAQS and WHO AQGs per Country

**FIGURE 3:**
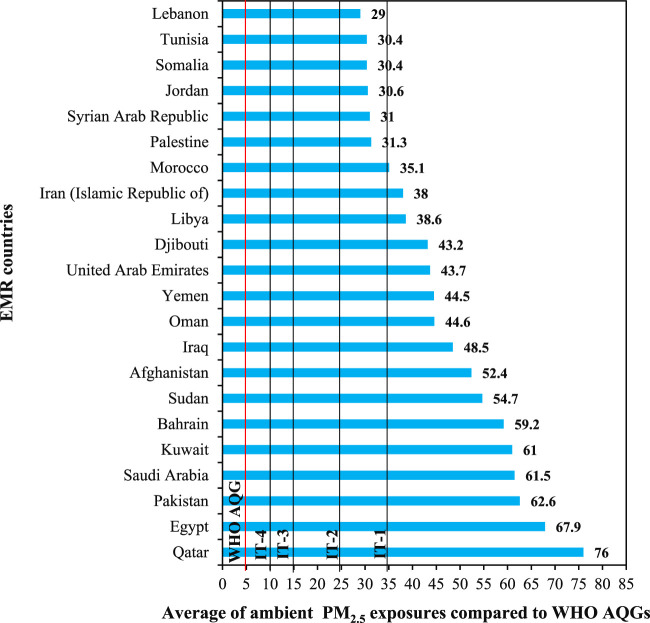
Average annual population-weighted fine particulate matter (PM_2.5_) exposure for 22 Eastern Mediterranean Region countries in 2019 estimated by Global Burden of Disease project compared to the updated World Health Organization Air Quality Guidelines and Interim Targets (Eastern Mediterranean Region. 2022).

Population weighted annual mean PM_2.5_ exposure levels in 2019, estimated by GBD project (17), were 6–15 times higher than WHO AQG level and, in 16 out of 22 countries in EMR, exceeded the highest of WHO ITs (IT-4, 35 μg m^−3^) (Figure 3). Reducing exposure to PM_2.5_ would, therefore, significantly reduce health impacts of the pollution. Based on the assumptions presented in the “methods” section, it can be expected that the reduction of mean exposure level to the AQG level (5 μg m^−3^) would be associated with all natural cause mortality in adults (age 30+) decreased by between 16.9% in Lebanon and 42.1% in Qatar (Table 1). Also the achievement of the IT-2 (25 μg m^−3^) would be connected with health benefits (between 3.0% in Lebanon and 37.5% in Qatar reduction of mortality).

Health benefits of the achievement of the NAAQS depends on both the NAAQS level and the current level of exposure in a country. The greatest benefits could be expected in Pakistan (30.7% decrease in mortality), where current PM_2.5_ levels are high and NAAQS relatively low (15 μg m^−3^). Smaller benefits (11.3%) can be expected in Jordan, where the NAAQS is the same as in Pakistan but PM_2.5_ exposure is ca. half of that in Pakistan. Achievement of more ambitious national standards (e.g., equal to WHO IT-4, 10 μg m^−3^) would result in a 14.73% reduction of mortality in Jordan. Nevertheless, since NAAQS levels are significantly higher than the AQG level, reduction of mortality attributed to particulate air pollution associated with the achievement of NAAQS for PM_2.5_ would reach between 34% (Egypt) and 92% (Sudan) of that expected for the achievement of AQG level in these countries.

Reduction of PM_2.5_ levels would be expected to result in lower age-adjusted air pollution-attributable mortality rates. However, the absolute number of attributable deaths in the future might not necessarily decrease due to changes in population size, age structure, and baseline cause- and age-specific mortality (23). Furthermore, it should be noted that the concentration-response function used to calculate PAFs is based on a meta-analysis of all globally available epidemiological studies, none of which was conducted in the EMR region where sand dust is a major contributor to PM_2.5_ exposure. Though we have assumed, based on current evidence, that this concentration-response function is applicable also in EMR, application of the “global” function in the region might affect precision of our estimates.

### Air Quality Management and Monitoring in EMR

Responses to “A questionnaire to collect health, population, and air quality monitoring and management data in the Eastern Mediterranean Region” were received from 12 out of 22 EMR countries (Afghanistan, Egypt, Iran, Iraq, Jordan, Kuwait, Lebanon, Morocco, Pakistan, Sudan, Tunisia and Yemen). Responses to the questions are summarized in Table 2. According to the survey, in eight of the 11 responding countries with a constitution, the right to clean air for the people is mentioned there. In most of these countries, also a national or subnational or multi-national/regional air quality action plan (AQAP) exists (the exceptions are Sudan and Yemen). The health component is included in the AQAP of six countries. In nine countries, the implementation of the actions is a shared responsibility of both the Department of Environment (DoE) and the Ministry of Health (MoH), while in Iraq and Pakistan just the DoE is responsible for AQAPs implementation. Such responsibilities are assigned also in Yemen, where no AQAP is formulated.

**TABLE 2:** Action plans on ambient air quality (Eastern Mediterranean Region. 2022).

Type of action	Present in countries												Not present	No response
	Afghanistan	Egypt	Iran	Iraq	Jordan	Kuwait	Lebanon	Morocco	Pakistan	Sudan	Tunisia	Yemen	Number of countries	
The right to clean air for the people specified in the constitution	x	x	x		x		x	x			x	x	3	11
National or subnational, or multi-national/regional air quality management strategy/framework/plan of action	x	x	x	x	x	x	x	x	x		x		2	10
Health component included in the AQAP			x		x	x	x	x			x		4	12
Dept. of Environment responsible for AQAP	x	x	x	x	x	x	x	x	x		x	x	0	11
Ministry of Health responsible for AQAP	x	x	x		x	x	x	x			x	x	2	11

A part of the WHO/CHE questionnaire was related to SDS and transboundary air pollution (TAP). Actions on these important sources of particulate matter air pollution were included in the AQAP and/or a subject of intergovernmental cooperation in two countries (Table 3). More common were the studies on the contribution of SDS and TAP to air pollution, reported from five countries. Early warning systems and/or other actions to reduce population exposure to SDS were implemented in four countries, also in those (Egypt and Jordan) missing comprehensive action plans on SDS or TAP. Specific control measures to control SDS in natural, rural, or urban areas were implemented in recent 5 years in Egypt, Jordan, and Kuwait. In two countries, health sector was involved in implementing the actions.

**TABLE 3:** Actions concerning sand and dust storm and transboundary air pollution (Eastern Mediterranean Region. 2022).

Type of action	Present in countries						Not present	No response
	Egypt	Iran	Iraq	Jordan	Kuwait	Lebanon	Number of countries	
Policy and/or action plan on SDS and TAP		x			x	x	9	10
Intergovernmental agreement/cooperation			x		x		10	10
Studies on contribution of SDS or TAP to air pollution	x	x	x		x	x	7	10
Early warning systems	x				x		10	10
Reducing exposure to SDS events	x	x		x	x		8	10
Control measures implemented in natural ecosystems in the recent 5 years				x	x		8	12
Control measures implemented in crop land in the recent 5 years	x			x	x		9	10
Control measures implemented in urban areas in the recent 5 years	x			x	x		7	12
Health sector involved in implementing and controlling SDS		x				x	7	13

An important element of air quality management is air quality monitoring, providing essential information on the magnitude of the air pollution problem as well as on the effectiveness of any actions undertaken to combat air pollution. Availability and accessibility of data from such monitoring in the EMR countries has been assessed through review of the most recent (released in April 2022) global air quality data base, created by WHO through gathering data directly from the member states as well through the search of a variety of publicly available sources (web pages, publications and reports). Before the data base is released, each member state is requested to review its contents and, if necessary, amend or correct the data related to this country. The data base contains information on annual mean concentration of PM_2.5_, PM_10_ and NO_2_ in 13 of the EMR countries (Supplementary Table S4), including Bahrain and UAE from which WHO/CHE has not received responses to its questionnaire survey. In most countries with data, the monitoring is available from a few cities only, but the coverage is much more extensive in Kuwait and Iran. Also in Egypt the number of monitoring locations is reported to be much larger (more than a 1000) but PM_10_ data are available in an aggregated form for just two regions (Delta Region and Greater Cairo). For most of the countries, the most recent data are from 2019 or even from 2020, indicating timely processing and publishing of the monitoring results. For the other countries, or from some cities, only older data are available. Completeness of the data is not always reported or shows that the annual mean is based on less than 75% of days in a year, decreasing the precision of the reported estimates of the pollution level.

### Recommendations for the EMR Countries

Reduction of population exposure to PM_2.5_ to the NAAQS level in the EMR countries where such standards have been established would bring significant health benefits. However, when we compare the set values of NAAQS for the classical ambient air pollutants in the countries of EMR with the WHO AQGs (2021) and its interim targets, it is evident that the standard values in these countries do not protect population health sufficiently. Therefore, it is important, from a population health perspective, to aim at lower air pollution levels than current standards in line with the recommendations of WHO AQGs, especially for PM_2.5_, e.g., by adopting time-specific goals for achievement of consecutive interim targets of the WHO AQGs (19–21, 24, 25). For instance, the countries of Iran, Saudi Arabia, and Pakistan with the 24-hour NAAQS values equal to 35 μg m^−3^ for PM_2.5_ could consider the WHO’s interim target-4 as their standards. To achieve this value, they would need to adopt and implement stricter air pollution abatement measures, particularly to tackle SDS as one of the most important sources of ambient air pollution in the EMR countries.

SDS are capable of transporting sediments over thousands of kilometres (26–28), meaning that this phenomenon has transboundary impacts. The EMR is one of the dustiest in the world due to the local dust sources and importantly its proximity to the Sahara Desert (26, 27). Windblown dust contributes ca. 51% of the mean population exposure to PM_2.5_ in North Africa and the Middle East, reaching 77% in Libya, 73% in Oman, and 66% in Kuwait and Morocco (29). Source-apportionment studies conducted in the EMR and reviewed by Faridi et al (2022) and a global assessment by McDuffie et al (2021), confirm a significant contribution of dust to PM_2.5_ levels in all countries in the region (14, 29). Nevertheless, other emission sources directly related to the combustion of fossil fuels contribute significantly as well. Energy production contributed ca. 12% of PM_2.5_ exposure in EMR, reaching 24% or 22% in Bahrain and Qatar, respectively. Road transport contribution was relatively smaller (7% on average in the region), reaching 11% in Iran (29). Studies reviewed in Faridi et al. (2022) also indicate a broad range of important PM sources, pointing also to a significance of secondary aerosols (29). Consequently, we believe that the use of source-specific air quality monitoring is needed to better specify sources of ambient PM_2.5_ and PM_10_ air pollution in SDS-countries and to address all of them in air quality management policy.

Concern regarding SDS is increasingly growing with regard to their huge impacts on human health, the environment, and even the economy (26, 30). Moreover, dealing with SDS and its far-reaching consequences has become one of the leading priorities within the global community and authoritative public health bodies (25, 27). By contrast, just two countries in the EMR (Iraq and Kuwait) have adopted intergovernmental cooperation actions to combat this important source in the region. Our analysis, based on the replies to WHO/CHE questionnaire and availability of air quality monitoring data, shows that the overall capacities of a large part of the EMR member states to cope effectively with air pollution, and in particular with SDS, are limited. Designing and implementing an integrated policy to deal with SDS and other pollution sources could improve ambient air quality in these countries. The detailed information on approaches to combat SDS is presented elsewhere (25–27). Designing and adopting an integrated coalition policy and/or action plan and cooperation agreement to tackle regional air pollution problems due to SDS events in the EMR countries supplementing the management and control of the local sources in each of these countries seems to be the most urgent task. Furthermore, using and following the scientific and well-documented solutions published by the global community and authoritative public health bodies would facilitate implementation of the actions in the region. Some of these publications are listed as Refs (25, 26, 30). Also section “4.4 Sand and dust storms” of the WHO global air quality guidelines contains relevant good practice statements on SDS (25).

### Conclusion

Improvement of air quality management, including international collaboration and prioritization of SDS, supported by an update (or establishment) of NAAQSs are essential elements for reduction of air pollution and its health effects in the EMR. Air quality monitoring, conducted with reliable methods and providing easily accessible data, facilitating identification of pollution sources, must be established or upgraded in most of the EMR countries to guide the actions and evaluate their effects.

## Author’s Note

This study is a part of a consultancy to provide a background assessment of air quality (AQ) management system for the Eastern Mediterranean countries in order to improve their capabilities to use the updated World Health Organization Air Quality Guidelines in compiling national AQ standards.
