# Supplementary material for: Ambient Air Quality Standards and Policies in Eastern Mediterranean Countries: A Review
Source: Int J Public Health. 2023 Feb 20;68:1605352. doi: 10.3389/ijph.2023.1605352 (PMC9986936; doi:10.3389/ijph.2023.1605352)
Supplement: Supplementary file 1 [file DataSheet1.docx]

**Ambient Air Quality Standards and Policies in Eastern Mediterranean Countries: A review^⋆^**

Sasan Faridi ^1, 2^, Michal Krzyzanowski ^3^, Aaron J. Cohen ^4, 5, 6^, Mazen Malkawi ^7^, Heba Adel Moh’d Safi ^7^, Fatemeh Yousefian ^8^, Faramarz Azimi ^9^, Kazem Naddafi ^1, 2^, Fatemeh Momeniha ^10^, Sadegh Niazi ^11^, Heresh Amini ^12^, Nino Künzli ^13, 14^, Mansour Shamsipour ^15^, Adel Mokammel ^2^, Vahid Roostaei ^2^, Mohammad Sadegh Hassanvand ^1, 2*^

^1^ Center for Air Pollution Research (CAPR), Institute for Environmental Research (IER), Tehran University of Medical Sciences, Tehran, Iran.

^2^ Department of Environmental Health Engineering, School of Public Health, Tehran University of Medical Sciences, Tehran, Iran.

^3^ Environmental Research Group, School of Public Health, Imperial College London, London, UK

^4^ Institute for Health Metrics and Evaluation, University of Washington, Seattle, Washington United States of America.

^5^ Boston University School of Public Health, Boston, Massachusetts, United States of America.

^6^ Health Effects Institute, Boston, Massachusetts, United States of America.

^7^ World Health Organization/ Regional Office of the Eastern Mediterranean/ Climate Change, Health and Environment Unit (WHO/EMR/CHE), Amman, Jordan.

^8^ Department of Environmental Health Engineering, Faculty of Health, Kashan University of Medical Sciences, Kashan, Iran.

^9^Environmental Health Research Center, School of Health and Nutrition, Lorestan University of Medical Sciences, Khorramabad, Iran.

^10^ Center for Solid Waste Research, Institute for Environmental Research (IER), Tehran University of Medical Sciences, Tehran, Iran.

^11^ Queensland University of Technology (QUT), Faculty of Science, School of Earth and Atmospheric Sciences, International Laboratory for Air Quality and Health, Brisbane, 4001, Australia.

^12^ Department of Public Health, University of Copenhagen, Copenhagen, Denmark.

^13^ Swiss Tropical and Public Health Institute, Allschwil, Switzerland.

^14^ University of Basel, Basel, Switzerland.

^15^ Department of Research Methodology and Data Analysis, Institute for Environmental Research (IER), Tehran University of Medical Sciences, Tehran, Iran.

**^⁎^** **Corresponding Author:** MS. Hassanvand, PhD, Center for Air Pollution Research (CAPR), Institute for Environmental Research (IER) and Department of Environmental Health Engineering, School of Public Health, Tehran University of Medical Sciences, Phone: +98 88978395, Fax: +98 88978397, 8^th^ Floor, No. 1547, North Kargar Avenue, Tehran, Iran ([hassanvand@tums.ac.ir](mailto:Hassanvand@tums.ac.ir)).

**^⋆^** This study is a part of a consultancy to provide a background assessment of air quality (AQ) management system for the Eastern Mediterranean countries in order to improve their capabilities to use the updated World Health Organization Air Quality Guidelines in compiling national AQ standards.

**2024** records identified (Scopus: **643**, PubMed: **33** and Web of Science: **50**) + 1297 records from Google Scholar database

**1083** titles screened

Full text reviewed (n = **5**)

**940** duplicates removed

Non-relevant records excluded (n = **1078**)

One paper used

No sufficient data (n = **4**)

**Figure S1**. Flowchart of search in the Scopus, PubMed, and Web of Science and Google Scholar database (Eastern Mediterranean Region. 2022).

**TABLE S1** Search results in the Scopus, PubMed, and Web of Science and Google Scholar database on the National Ambient Air Quality Standards and the World Health Organization Air Quality Guidelines (Eastern Mediterranean Region. 2022).

| Study ID | Title of paper | Journal |
| --- | --- | --- |
| (Krzyzanowski and Cohen 2008) | Update of WHO air quality guidelines | Air Quality, Atmosphere, and Health |
| (Vahlsing and Smith 2012) | Global review of national ambient air quality standards for PM_10_ and SO_2_ (24 h) | Air Quality, Atmosphere, and Health |
| (Joss, Eeftens et al. 2017) | Time to harmonize national ambient air quality standards | International Journal of Public Health |
| (Amini 2021) | WHO Air Quality Guidelines Need to be Adopted | International Journal of Public Health |
| (Hoffmann, Boogaard et al. 2021) | WHO Air Quality Guidelines 2021–Aiming for Healthier Air for all: A Joint Statement by Medical, Public Health, Scientific Societies and Patient Representative Organisations | International Journal of Public Health |

**TABLE S2** Number of documents identified by hand-searching (Eastern Mediterranean Region. 2022).

| Countries | Number of documents explored (Air Quality Policies and the findings of a research that UNEP conducted in 2015) |
| --- | --- |
| Afghanistan | 3 |
| Bahrain | 1 |
| Djibouti | 1 |
| Egypt | 2 |
| Islamic Republic of Iran | 2 |
| Iraq | 2 |
| Jordan | 2 |
| Kuwait | 2 |
| Lebanon | 3 |
| Libya | 1 |
| Morocco | 2 |
| Oman | 2 |
| Pakistan | 2 |
| Palestine | 0 |
| Qatar | 2 |
| Saudi Arabia | 2 |
| Somalia | 1 |
| Sudan | 1 |
| Syrian Arab Republic | 2 |
| Tunisia | 2 |
| United Arab Emirates | 2 |
| Yemen | 1 |
| At Global scale^[[1]](#footnote-1)^ | 2 |

**TABLE S3** Ambient air quality standards in the Eastern Mediterranean Region countries and comparison with the World Health Organization Air Quality Guidelines (Eastern Mediterranean Region. 2022).

| **Country** | **(mg/m^3^)** | | **(µg/m^3^)** | | | | | | | | | | | |
| --- | --- | --- | --- | --- | --- | --- | --- | --- | --- | --- | --- | --- | --- | --- |
|  | **CO** | | **NO_2_** | | | **SO_2_** | | | **O_3_** | | **PM_2.5_** | | **PM_10_** | |
|  | **1-hr** | **8-hr** | **1-hr** | **24-hr** | **Annual** | **1-hr** | **24-hr** | **Annual** | **1-hr** | **8-hr** | **24-hr** | **Annual** | **24-hr** | **Annual** |
| Afghanistan | 30 | 10 | - | 80 | 40 | - | 50 | - | - | 100 | 75 **^a^** | 35 | 150 **^a^** | 70 |
| Bahrain | 20 | 10 | 200 | 150 | 40 | 300 | 125 | 50 | - | 150 | 50 | 25 | 340 | 80 |
| Egypt | 30 | 10 | 300 | 150 | 60 | 300 | 125 | 50 | 180 | 120 | 80 | 50 | 150 | 70 |
| Iran | 40 | 10 | 200 | - | 100 | 196 | 395 | - | - | 159 | 35 **^b^** | 12 | 150 **^b^** | - |
| Iraq | 43 | 11 | 200 | 100 | 40 | 423 | 169 | 56 | 211 | 127 | 25 | 10 | 100 | 50 |
| Jordan | 26 | 9 | 210 | 80 | 50 | 300 | 140 | 40 | 120 | 80 | 65 **^c^** | 15 | 120 **^c^** | 70 |
| Kuwait | 40 | - | 190 | - | 40 | 196 | 50 | - | - | 150 | 75 **^d^** | - | 350 **^d^** | - |
| Lebanon | 30 | 10 | 200 | 150 | 100 | 350 | 120 | 80 | 150 | 100 | - | - | 80 | - |
| Libya | 30 | 10 | 400 | 150 | - | 350 | 150 | 60 | 200 | 120 | - | - | 150 | - |
| Morocco | - | 10 | 200 | - | 50 | - | 125 | 20 | - | - | - | - | 50 | - |
| Oman | 30 | 10 | 250 | 130 | - | 350 | 150 | - | - | 120 | 65 | - | 150 | - |
| Pakistan | 10 | 5 | - | 80 | 40 | - | 120 | 80 | 130 | - | 35 **^e^** | 15 | 150 **^e^** | 120 |
| Palestine | 26 | 9 | 210 | 80 | 50 | 300 | 140 | 40 | 120 | 80 | - | - | 120 **^d^** | 70 |
| Qatar | 40 | 10 | 400 | 150 | 100 | - | 365 | 80 | 235 | 120 | - | - | 150 | 50 |
| Saudi Arabia | 40 | 10 | 200 | - | 100 | 441 | 217 | 65 | - | 157 | 35 **^f^** | 15 | 340 **^f^** | 50 |
| Sudan | 26 | 9 | 220 | 80 | 50 | 170 | 140 | 31 | - | 60 | 25 | 10 | 125 **^g^** | 73 |
| Syria | 30 | 10 | 200 | - | 40 | 350 | 125 | 50 | 160 | 120 | - | - | 100 | 50 |
| Tunisia | 40 | 10 | 660 | - | 200 | - | 365 | 80 | 235 | - | - | - | 260 | 80 |
| UAE^*^ | 30 | 10 | 400 | 150 | - | 350 | 150 | 60 | 200 | 120 | - | - | 150 | - |
| Djibouti | **These countries have not set standard for the specific pollutant/ pollutants, or without any information available**. | | | | | | | | | | | | | |
| Somalia |  |  |  |  |  |  |  |  |  |  |  |  |  |  |
| Yemen |  |  |  |  |  |  |  |  |  |  |  |  |  |  |
| WHO AQGs-2021 | 35 | 10 | 200 | 25^d^ | 10 | - | 40^d^ | - | - | 100^d^ | 15^d^ | 5 | 45^d^ | 15 |

The number of days per year is allowed that the pollutant levels exceeded standards: **a)** 18 days/year (24 hourly values should be met 95% of the year); **b)** 4 days/year **c)**; 3 days/year **d**) 3 days/year (24 hourly values should be met 99% of the year) ; **e**) 7 days/year (24 hourly values should be met 98% of the in a year. 2% of the time, but not on two consecutive days); **f)** 12 days/year; **g)** 3 days/month.

* United Arab Emirates (UAE).

**TABLE S4** Data from particulate matter (PM_2.5_, PM_10_) and Nitrogen dioxide (NO_2_) monitoring in Eastern Mediterranean Region countries in 2015-2020 available in the World Health Organization 2022 Air Quality database[5] (Eastern Mediterranean Region. 2022).

| **Country** | **Number of cities with data on** | | | **Last year with data on** | | **Comments** |
| --- | --- | --- | --- | --- | --- | --- |
|  | **PM_2.5_** | **PM_10_** | **NO_2_** | **PM_2.5_ / PM_10_** | **NO_2_** |  |
| Afghanistan | 1 | - | - | 2019 / - | - | (a), (b) |
| Bahrain | 3 | 3 | 2 | 2019 / 2019 | 2019 | - |
| Egypt | - | 2 | - | - / 2015 | - | (c) (h) |
| Iran | 55 | 1 | 1 | 2016 / 2016 | 2016 | (c) |
| Iraq | 2 | 3 | 3 | 2019 / 2020 | 2020 | (d) |
| Jordan | 2 | 5 | 4 | 2019 / 2019 | 2019 | (e) (f) |
| Kuwait | 11 | 16 | 11 | 2020 / 2020 | 2020 | (f) |
| Lebanon | 1 | 1 | - | 2016 / 2016 | - | (b) |
| Morocco | - | 11 | 6 | - / 2020 | 2020 | (d) (f) |
| Pakistan | 4 | - | 1 | 2020 / - | 2020 | (b) (d) (f) (g) |
| Qatar | 3 | 3 | - | 2016 / 2016 | - | (c) |
| Saudi Arabia | 2 | 1 | - | 2019 / 2017 | - | (b) |
| UAE | 2 | 6 | 6 | 2019 / 2020 | 2020 | (c) |

1. Data from the U.S. Department of State (AQ monitoring by U.S. Embassy)
2. Completeness of data: less than 75% of days/year (in all or some cities)
3. Completeness of data not specified in the WHO DB
4. Completeness of data specified for some cities only
5. Data on PM_2.5_ incomplete
6. For some locations, data from earlier years of the period 2016-2020 available only
7. Data from the U.S. Department of State (AQ monitoring by U.S. Embassy) for some cities
8. Results in WHO DB based on measurements in >1000 stations

**Questionnaire S1.**

**A questionnaire to collect health, population, and air quality monitoring and management data for assessing the capacities in the (Insert the name of responding country)**

**This questionnaire has three overall sections to collect air quality monitoring and management data as well as health-based data for assessing the capacities in the Eastern Mediterranean Region against air pollution and its health consequences. Your collaboration can be effective to help the better adoption and implementation of air pollution control abatements to this leading environmental risk factors in this region.**

**Personal Information**

Who is responding to the questionnaire?

| Full Name: |  |
| --- | --- |
| Country: |  |
| State/Province/City: |  |
| Ministry/Department: |  |
| Professional Title: |  |
| Email Address: |  |
| *Note that this information will be used in developing a technical paper and a plan of action for WHO/EMR/CEH and will not be published upon approval of the responders; it will only be used for decision-making purposes in order to improve the capabilities of the Eastern Mediterranean Region against air pollution and its health consequences. | |

**Ambient Air Quality Management**

| **1. Is there a national or subnational or multi-national/regional air quality management strategy/framework/plan of action in your country?**  Yes  No  **If yes, is the health component included in the strategy/framework/plan of action?** |
| --- |
| **(Hereunder are pop-up questions if your answer to question 1 is Yes)**  **1.1 Provide links to access the strategy/framework/plan of action, or provide/attach these documents as pdf files.**   \| URL link \|  \| \| --- \| --- \| |
| **1.2 Does the constitution of your country recognize the right to clean air?**  Yes  No |
| **1.3 Quote the article of the constitution in which we can find reference to right to clean air**   \|  \| \| --- \| |
| **1.4 Which institutions are responsible for implementing the air quality management strategy/framework/plan of action in your country?**   \|  \| \| --- \| |
| **1.5 How is the strategy/framework/plan of action implemented? (Select all that apply)**  National air quality action plan  Sectoral plans  A clean air Act  Other (Please specify): |
| **1.6 Are there clean air action plans at subnational levels (for regions and/or cities)?**  Yes  No |
| **(Conditional question if 1.6 is Yes)**  **1.6.1 Which regions and/or cities? Please add a link to each action plan or attach it.**   \|  \| \| --- \| |

**Ambient Air Quality Standards**

| **1. Does your country have national ambient air quality standards?**  Yes  No  **If yes, Are the national standards based on the WHO Air Quality Guidelines or any other health-based standards?** |
| --- |
| **(Hereunder are pop-up questions if your answer to question 1 is Yes)**  **1.1 Provide links to access the national ambient air quality standards, or provide/attach these documents as pdf files.**   \| URL link \|  \| \| --- \| --- \| |
| **1.2 Which ambient air pollutants are regulated?**  PM_10_  PM_2.5_  Ozone (O_3_)  Nitrogen Dioxide (NO_2­_)  Sulphur Dioxide (SO_2­_)  Carbon Monoxide (CO)  Other (Please specify): |
| **1.3 Are these air quality standards legally enforceable?**  Yes, legally enforceable through laws, regulations or other similar instruments.  No, recommended guidelines with no legal force. |
| **(Conditional question if 1.3 is Yes)**  **1.4.1 List below the institutions responsible for enforcement of standards.**   \|  \| \| --- \| |

**Ambient Air Quality Monitoring**

| **1. Does your country have a national/subnational ambient air quality monitoring network?**  Yes, continuous monitoring at multiple sites  Yes, monitoring done periodically at a few sites  Yes, a monitoring network exists but is not operational  No monitoring |
| --- |
| **(Hereunder are pop-up questions if your answer to question 1 is Yes, network exists but not operational)**  **1.1 Why it is not operational?** – Let us know what are the challenges and problems that prevent a fully operational monitoring network (e.g., outdated infrastructure, lack of maintenance, etc.)**.**   \|  \| \| --- \| |
| **(Hereunder are pop-up questions if your answer to question 1 is Yes)**  **1.2 Which type of monitoring is used in the network? (Select all that apply)**  Stationary reference monitors  Mobile reference monitors  Hybrid network with both stationary and mobile reference monitors  Passive samplers  Low-cost sensors  Other (Please specify): |
| **(Hereunder are pop-up questions if your answer to question 1.2 is stationary reference monitors)**  **1.2.1 Has air quality monitoring site selection process for stationary reference monitors been made based on a specific guideline?**  Yes  No  **1.2.2 Please specify which guideline and provide its link, or provide/attach these documents as pdf files.**   \| URL link \|  \| \| --- \| --- \| \|  \| \| |
| **1.3 Which ambient air pollutants are monitored?**  PM_10_  PM_2.5_  Ozone (O_3_)  Nitrogen Dioxide (NO_2­_)  Sulphur Dioxide (SO_2­_)  Carbon Monoxide (CO)  Other (Please specify): |
| **1.4 Which institutions are responsible for ambient air quality monitoring?**   \|  \| \| --- \| |
| **1.5 Is there a specific guideline to make quality assurance/quality control for ambient air quality data in your country?**  Yes  No |
| **1.6 Do the responsible institutions use the guideline for quality assurance/quality control in your country?**  Yes  No |
| **(Conditional question if 1.6 is Yes)**  **1.6.1 Please specify which guidelines and provide its/their links, or provide the guidelines if possible.**   \|  \| \| --- \| |
| **1.7 How is access to ambient air quality data?**  Web-based  Offline |
| **(Conditional question if 1.7 is web-based)**  **1.7.1 Provide the links for ambient air quality data (national and subnational level), or approach how the researcher/ investigator can get/request the data.**   \| URL link \|  \| \| --- \| --- \| |
| **1.8 Please specify the data coverage for ambient air monitoring stations. (For example, hourly data coverage for station A in the city X was in the range of 50-75%, or >75% in a given year).**   \| PM_10_ \|  \| \| --- \| --- \| \| PM_2.5_ \|  \| \| O_3_ \|  \| \| NO_2­_ \|  \| \| SO_2­_ \|  \| \| CO \|  \| \| Other (Please specify): \|  \| |
| **1.9 Please specify which regions and/or cities have air quality monitoring stations.**   \|  \| \| --- \|   **1.9.1 Please report the number of air quality monitoring stations in each region, and mention the type of them (for example, Traffic, Industrial, Residential, Background, and Special).**   \|  \| \| --- \| |

**Air Pollution Sources: Source apportionment/Emission inventory Studies**

| **1. Have been conducted any national or subnational air pollution source apportionment/emission inventory studies in your country to date?**  Yes  No |
| --- |
| **(Hereunder are pop-up questions if your answer to question 1 is Yes)**  **1.1 Briefly describe the summary of the source apportionment/emission inventory study (study location, date, method, and share of sources) has been done to date.**   \|  \| \| --- \|   **1.2 Provide links to access the source apportionment/emission inventory studies, or provide/attach these studies as pdf files.**   \| URL link \|  \| \| --- \| --- \| |

**Air Pollution Sources: Transportation**

| **1. Are there national/subnational vehicle emission/fuel standards in your country?**  Yes  No  **If yes, are the standards based on/ includes health impact component?** |
| --- |
| **(Hereunder are pop-up questions if your answer to question 1 is Yes)**  **1.1 Provide links to access the transport standards, or provide/attach these standards as pdf files.**   \| URL link \|  \| \| --- \| --- \| |
| **1.2 Specify whether the standards are (for example Euro 3, 4, 5/6, etc.).**   \|  \| \| --- \| |
| **1.3 Are fuel quality standards in place?**  Yes  No |
| **1.4 What is the maximum allowable sulfur level in petrol, by law?**  0-50 ppm  51-500 ppm  above 500 ppm |
| **1.5 What is the maximum allowable sulfur level in diesel, by law?**  0-50 ppm  51-500 ppm  above 500 ppm |
| **1.6 Does your country have an age limit or ban on used vehicle imports?**  Ban on all used vehicle imports  Allows only import of vehicles less than 5 years  Allows import of vehicles above 5 years |
| **1.7 Which of the following measures have been implemented in the past 5 years?**  Adoption and enforcement of advanced vehicle emission standards (e.g. Euro 5/6)  Provision of access to public transport and non-motorized transport infrastructure  Adoption of cleaner fuel import and production standards  Vehicle inspection and maintenance programs  Promotion of electric mobility  Other (Please specify):  No measures implemented  **1.8 Are the national/subnational vehicle emission/fuel standards implemented?**  Yes  No  **(Hereunder is pop-up question if your answer to question 1.8 is No)**  **1.8.1 Please describe the reasons**   \|  \| \| --- \| |
| **(Subsidiary question if any measure is selected in 1 above)**  **1.9 How is the impact of the above measured?**  Continuous air quality monitoring  Source apportionment studies  Air quality modelling and scenario analysis  National emissions inventory for the transport sector  Cost benefit analysis  Health impact assessments  Percentage of vehicle failure rate to emission standards  Other (Please specify): |

**Air Pollution Sources: Industrial emissions**

| **1. Are there incentives and/or mandatory policies in your country to promote cleaner production, energy efficiency and pollution abatement for industries?**  Yes  No  **If yes, are the standards based on/ includes health impact component?** |
| --- |
| **(Hereunder are pop-up questions if your answer to question 1 is Yes)**  **1.1 Briefly describe the incentives and/or policies from your country or provide the links of them.**   \|  \| \| --- \| |
| **1.2 Are national emission standards for industries contained in laws or regulations?**  Yes  No |
| **1.3 Provide the links for the standards, or provide/attach these standards as pdf files.**   \| URL link \|  \| \| --- \| --- \| |
| **1.4 Which of the following measures have been implemented in the past 5 years?**  Increased investment in renewable energy and energy efficiency  Industrial emission standards and regulations  Use of environmental impact assessments to regulate industries  Policies on efficient resource use (fuel and electricity)  Policies and legal frameworks to promote cleaner production  Other (Please specify):  No measures implemented |
| **(Subsidiary question if any measure is selected in 1 above)**  **1.5 How is the impact of the above measured?**  Continuous air quality monitoring  Source apportionment studies  Air quality modelling and scenario analysis  Emission inventory for the industrial sector  Cost benefit analysis  Health impact assessments  Other (Please specify): |

**Air Pollution Sources: Natural sources (sand and dust storm (SDS) events) and transboundary pollution**

| **1A. Is there a policy and/or action plan in your country to combat SDS and prevent transboundary pollution?**  Yes  No  **If yes, Is the health sector involved in implementing and controlling these policies/ plans?**  **(Hereunder are pop-up questions if your answer to question 1A is Yes)**  **1A.1 Briefly describe the national policies, measures and/or action plan from your country.**   \|  \| \| --- \|   **1A.2 Provide the links for the national policies, measures and/or action plan in your country, or provide/attach these documents as pdf files.**   \| URL link \|  \| \| --- \| --- \| |
| --- | --- | --- | --- |
| **1B. Is there a coalition policy and/or action plan (an intergovernmental agreement/cooperation to tackle regional air pollution problems due to SDS events or transboundary pollution) with other countries to these issues in your country?**  Yes  No  **(Hereunder are pop-up questions if your answer to question 1B is Yes)**  **1B.1 Briefly describe the national policies, measures and/or action plan from your country.**   \|  \| \| --- \|   **1B.2 Provide the links for an intergovernmental agreement/cooperation to tackle regional air pollution problems due to SDS events with middle east countries to combat SDS and prevent transboundary pollution, or attach these documents as pdf files.**   \| URL link \|  \| \| --- \| --- \|   **2. Is there a given study/studies to reveal the contribution of SDS events or transboundary pollution on ambient air pollution in your country?**  Yes  No  **(Hereunder are pop-up question if your answer to question 2 is Yes)**  **2.1 Provide links to access that studies, or briefly describe the share of these sources.**   \| URL link \|  \| \| --- \| --- \| \|  \| \|   **3. Is there any early warning system for forecasting and communicating the SDS events or transboundary pollution?**  Yes  No  **(Hereunder are pop-up question if your answer to question 3 is Yes)**  **3.1 Provide links to access the early warning system for forecasting the SDS events/transboundary pollution, or briefly describe the early warning system**   \| URL link \|  \| \| --- \| --- \| \|  \| \|   **4. Are there any actions for reducing the exposure of people to the SDS events/transboundary pollution in your country?**  Yes  No  **If yes, is the health sector involved in implementing and raising these actions?**  **(Hereunder are pop-up questions if your answer to question 4 is Yes)**  **4.1 Briefly describe the actions for reducing the exposure of people to the SDS events/transboundary pollution in your country.**   \|  \| \| --- \|   **5. Which of the following measures have been implemented at national level in the past 5 years?**  Control measures in natural ecosystems and rangelands  Control measures in crop land  Control measures in industrial settings  Protection of urban areas and infrastructure  Integrated control strategies  Establishing monitoring, prediction and warning systems for SDS/transboundary pollution  Other (Please specify):  No measures implemented  **6. Which of the following measures have been implemented at intergovernmental level in the past 5 years?**  Establishing intergovernmental agreement to tackle regional problems of SDS events/transboundary pollution  Control measures in crop land  Control measures in industrial settings  Protection of urban areas and infrastructure  Integrated control strategies  Establishing monitoring, prediction and warning systems for SDS/transboundary pollution  Other (Please specify):  No measures implemented |
| **(Subsidiary question if any measure is selected in 5 and 6 above)**  **7. How is the impact of the above measured?**  Continuous air quality monitoring  Source apportionment studies  Air quality modelling and scenario analysis  Cost benefit analysis  Health impact assessments  Other (Please specify): |

**Air Pollution Sources: Commercial emissions**

| **1. Are there incentives or mandatory policies in your country to promote emissions abatement for commercial sectors?**  Yes  No  **If yes, is health component involved in these incentives and/or policies.** |
| --- |
| **(Hereunder are pop-up questions if your answer to question 1 is Yes)**  **1.1 Briefly describe the incentives and/or policies from your country.**   \|  \| \| --- \| |
| **1.2 Are national emission standards for commercial sectors contained in laws or regulations?**  **Yes**  **No** |
| **1.3 Provide the links for the standards, or provide as pdf files.**   \| URL link \|  \| \| --- \| --- \| |
| **1.4 Which of the following measures have been implemented in the past 5 years?**  Increased investment in cleaner approaches  Commercial emission standards and regulations  Policies and legal frameworks to promote pollution control devices used by commercial sectors  Other (Please specify):  No measures implemented |
| **(Subsidiary question if any measure is selected in 1 above)**  **1.5 How is the impact of the above measured?**  Continuous air pollutants monitoring  Source apportionment studies  Air quality modelling and scenario analysis  Emission inventory for the commercial sector  Cost benefit analysis  Health impact assessments  Other (Please specify): |

**Air Pollution Sources: Construction emissions**

| **1. Are there incentives or mandatory policies in your country to promote emissions abatement for construction activities?**  Yes  No  **If yes, is health component involved in these incentives and/or policies.** |
| --- |
| **(Hereunder are pop-up questions if your answer to question 1 is Yes)**  **1.1 Briefly describe the incentives and/or policies from your country [No word limit].**   \|  \| \| --- \| |
|  |
| **1.2 Provide the links for the incentives and/or policies, or provide them as pdf files**   \| URL link \|  \| \| --- \| --- \| |
| **1.3 Which of the following measures have been implemented in the past 5 years?**  Enclosing construction sites  Covering demolition waste and stacked materials  Covering bare lands  Cleaning vehicles entering and leaving constructon sites  Wet excavation  Enclosing demolition waste transport vehicles  Other (Please specify): |
| **(Subsidiary question if any measure is selected in 1.3 above)**  **1.3.1 How is the impact of the above measured?**  Continuous air pollutants monitoring  Source apportionment studies  Air quality modelling and scenario analysis  Emission inventory  Cost benefit analysis  Health impact assessments  Other (Please specify): |

**Air Pollution Sources: Solid Waste Management**

| **1. Is open burning of agricultural, municipal and/or other forms of solid waste prohibited by law or provisions in the national legislation of your country?**  Yes, burning of solid waste is strictly regulated  Yes, burning of solid waste is regulated but still practiced  No, burning of solid waste is not regulated and is practiced |
| --- |
| **(Hereunder are pop-up questions if your answer to question 1 is Yes)**  **1.1 Provide links to access the management of solid waste regulations and policies, or provide them as pdf files.**   \| URL link \|  \| \| --- \| --- \| |
| **1.2 Briefly describe the programs and incentives from your country for solid waste management.**   \|  \| \| --- \| |
| **1.3. Which of the following measures have been implemented in the past 5 years?**  Implementation of national or urban solid waste management plans  Formulation and enforcement of solid waste management regulations  Improved collection, separation and environmentally sound disposal of solid waste  Regulating open burning of solid waste  Landfill gas recovery  Campaigns for behavioral change  Other (Please specify):  No measures implemented |
| **(Subsidiary question if any measure is selected in 1.3 above)**  **1.3.1 How is the impact of the above measured?**  Continuous air quality monitoring  Source apportionment studies  Air quality modelling and scenario analysis  Emission inventory for the waste sector  Cost benefit analysis  Health impact assessments  Other (Please specify): |

**Air Pollution Sources: Residential (Household Air Pollution)**

| **1. Are there national programs that promote use of clean energy in households for cooking and heating in your country?**  Yes  No |
| --- |
| **(Hereunder are pop-up questions if your answer to question 1 is Yes)**  **1.1 Briefly describe the programs from your country.**   \|  \| \| --- \| |
| **1.2 Which are the dominant fuels for cooking and heating in your country?**  Natural gas  Propane/LPG  Electricity  Coal/Charcoal  Wood/Biomass  Agricultural crop waste  Other (Please specify): |
| **1.3 Which of the following measures have been implemented in the past 5 years?**  Adoption of low-emission cooking stoves and fuels  Expanding use of Liquid Petroleum Gas (LPG)  Increased energy efficiency in housing  Improve access to green technologies for residential heating  Other (Please specify):  No measures implemented |
| **(Subsidiary question if any measure is selected in 1.3 above)**  **1.3.1 How is the impact of the above measured?**  Continuous air quality monitoring  Source apportionment studies  Air quality modelling and scenario analysis  Emissions inventory  Cost benefit analysis  Health impact assessments  Other (Please specify): |

**Air Pollution Sources: Agriculture**

**As we all know, the agricultural sector is a major source of ammonia-induced particulate matter and methane (the main precursor of tropospheric ozone) from manure and inorganic fertilizer application.**

| **1. Does your country have incentive or mandatory policies to promote sustainable agricultural practices (such as agriculture waste management, livestock manure management and use of organic fertilizers)?**  Yes  No |
| --- |
| **(Hereunder are pop-up questions if your answer to question 1 is Yes)**  **1.1 Briefly describe the programs and incentives from your country.**   \|  \| \| --- \| |
| **2. Which of the following measures have been implemented in the past 5 years?**  Providing alternatives for open burning of agricultural waste  Closed storage and improved livestock manure management  Reduced food waste  Methane capture for energy use  Other (Please specify):  No measures implemented |
| **(Subsidiary question if any measure is selected in 1 above)**  **2.1. How is the impact of the above measured?**  Continuous air quality monitoring  Source apportionment studies  Air quality modelling and scenario analysis  Emission inventory  Cost benefit analysis  Health impact assessments  Other (Please specify): |

**Availability and accessibility of health-based data**

| **1A. Are population statistics available at the national, district/region or city level?**  Yes  No |
| --- |
| **(Hereunder are pop-up questions if your answer to question 1A is Yes)**  **1A.1 Are population statistics regularly updated for the available data?**  Yes  No  **2A. Is population distribution by age group available at the national, district/region or city level?**  Yes  No  **3A. Could you please provide a URL link access (for Q1 &Q2), or approach how can the researcher/ investigator get/request this type of data.**   \| URL link \|  \| \| --- \| --- \| \|  \| \|   **1B. Are baseline mortality rates (all cause and cause specific deaths) available at the national, district/region or city level?**  Yes  No  **(Hereunder are pop-up questions if your answer to question 1B is Yes)**  **1B.1 Are these data regularly updated?**  Yes  No  **2B. Are these data by age group available at the national, district/region or city level?**  Yes  No  **3B. Could you please provide a URL link access (for Q1B &Q2B), or approach how can the researcher/ investigator get/request this type of data.**   \| URL link \|  \| \| --- \| --- \| \|  \| \|   **1C. Is the number of deaths from all-natural causes, age 25+ available at the national, subnational, regions/district or city level?**  Yes  No  **2C. Is the number of deaths related to Ischemic heart disease (IHD), age 25+ available at the national, subnational, region, or city level?**  Yes  No  **3C. Is the total number of deaths from cardiovascular diseases (CVD), all ages available at the national, subnational, regions/district or city level?**  Yes  No  **4C. Is the total number of deaths from respiratory diseases, all ages available at the national, subnational, regions/district or city level?**  Yes  No  **5C. Is the number of deaths related to lung cancer (LC), age 25+ available at the national, subnational, region, or city level?**  Yes  No  **6C. Is the number of deaths related to chronic obstructive pulmonary disease (COPD), age 25+ available at the national, subnational, region, or city level?**  Yes  No  **7C. Is the number of deaths related to lower respiratory infections (LRIs), age 25+ available at the national, subnational, region, or city level?**  Yes  No  **8C. Is the number of deaths related to cerebrovascular disease (stroke), age 25+ available at the national, subnational, region, or city level?**  Yes  No  **9C. Is the number of deaths related to acute lower respiratory infections (ALRI), age 0-5 available at the national, subnational, region, or city level?**  Yes  No  **10C. Are hospital admissions for cardiovascular diseases (CVD) available at the national, subnational, regions/district or city level?**  Yes  No  **11C. Are hospital admissions for respiratory diseases available at the national, subnational, regions/district or city level?**  Yes  No  **(Hereunder are pop-up questions if your answer to question 1C to 11C is Yes)**  **How can the researcher/investigator get/request these data, please provide a URL link (for each question/type) or provide an approach for getting/requesting these data.**   \| URL link \|  \| \| --- \| --- \| \|  \| \| |

**Information Release and Public Participation**

**As we all know information is power. Data transparency regarding all issues of air pollution enables all elements of society to participate in achieving air pollution abatement measures.**

| **1A. Does your country have a regular program/plan/policy to inform people regarding air quality status and its impacts at national or subnational level?**  Yes  No |
| --- |
| **(Hereunder are pop-up questions if your answer to question 1 is Yes)**  **1A.1 Briefly describe the programs from your country.**   \|  \| \| --- \| |
| **1B. Does your country have a regular program/plan/policy to increase the public awareness and their role in improving air quality status and its beneficial effects at national or subnational level?**  Yes  No |
| **(Hereunder are pop-up questions if your answer to question 1B is Yes)**  **1B.1 Briefly describe the programs from your country.**   \|  \| \| --- \| |

**Human Resources and Institutional Capabilities**

| **1. Do you know a given expert group or a governmental/ nongovernmental institution to investigate on various issues of air pollution in your country?**  Yes  No |
| --- |
| **(Hereunder are pop-up questions if your answer to question 1 is Yes)**  **1.1 Please mention them.**   \|  \| \| --- \| |

**Self-Report section**

| **What are the challenges your country face, and what obstacles are preventing assistance in improving air quality (indoor and outdoor) or meeting WHO AQ guidelines (Please mention those have been omitted in this questionnaire)?** |
| --- |

**Acknowledgement**

Thank you for taking time to complete this questionnaire. Your response is crucial to us and will contribute to our analysis of the top actions that lead to improved air quality in the **(insert the name of responding country)**.

We wish to thank you once again for your valuable time and generous participation in this effort. **Note:** To design this questionnaire, we have used mainly the document entitled “UNEP ‘Actions on Air Quality’ Report Update: Questionnaire to Member States” (<https://wedocs.unep.org/handle/20.500.11822/32833>) developed by Soraya Smaoun and Victor Nthusi in United Nations Environment Programme (2020), though we have added some questions to their questionnaire.

**Questions and/or clarifications can be emailed to those mentioned above Mohammad Sadegh Hassanvand (**[**hassanvand@tums.ac.ir**](mailto:hassanvand@tums.ac.ir)**; mshasanvand@gmail.com) and Sasan Faridi (**[**sfaridi@sina.tums.ac.ir**](mailto:s-faridi@razi.tums.ac.ir)**; sasanfaridi67@gmail.com).**

**References**

Amini, H. (2021). "WHO Air Quality Guidelines Need to be Adopted." International journal of public health **66**.

Hoffmann, B., H. Boogaard, A. de Nazelle, Z. J. Andersen, M. Abramson, M. Brauer, B. Brunekreef, F. Forastiere, W. Huang and H. Kan (2021). "WHO Air Quality Guidelines 2021–Aiming for Healthier Air for all: A Joint Statement by Medical, Public Health, Scientific Societies and Patient Representative Organisations." International Journal of Public Health **66**.

Joss, M. K., M. Eeftens, E. Gintowt, R. Kappeler and N. Künzli (2017). "Time to harmonize national ambient air quality standards." International Journal of Public Health **62**(4): 453-462.

Krzyzanowski, M. and A. Cohen (2008). "Update of WHO air quality guidelines." Air Quality, Atmosphere & Health **1**(1): 7-13.

Vahlsing, C. and K. R. Smith (2012). "Global review of national ambient air quality standards for PM 10 and SO 2 (24 h)." Air Quality, Atmosphere & Health **5**(4): 393-399.

1. Air Pollution Series Actions on Air Quality: A Global Summary of Policies and Programmes to Reduce Air Pollution"(<https://www.unep.org/resources/report/actions-air-quality-global-summary-policies-and-programmes-reduce-air-pollution>) and "Regulating Air Quality The first global assessment of air pollution legislation" (<https://wedocs.unep.org/bitstream/handle/20.500.11822/36666/RAQ_GAAPL.pdf>) [↑](#footnote-ref-1)
